# Cellular and molecular characteristics of blastoderm adhesion of the chick embryo

**DOI:** 10.5713/ab.260296

**Published:** 2026-06-15

**Authors:** Yuna Jo, Ju Hyeon Kim, Hyung Chul Lee

**Affiliations:** 1 School of Biological Sciences and Technology, College of Natural Sciences, Chonnam National University, Gwangju, Korea; 2 Institute of Plant Biomaterials and Biomedical Science, Chonnam National University, Gwangju, Korea

**Keywords:** Blastoderm, Chick Embryo, Epiboly, Vitelline Membrane

## Abstract

**Objective:**

During early avian development, the blastoderm border must adhere to the vitelline membrane (VM) in order to spread tissue evenly. Classical studies have demonstrated that blastoderm edge tissue possesses intrinsic adhesive properties to the VM; however, the spatial extent, developmental changes, and molecular characteristics of this adherence remain poorly understood. This study aimed to characterize the molecular and functional properties of blastoderm adhesion to the VM.

**Methods:**

Explant assays were performed using chick embryos at different developmental stages. Tissues were isolated from defined blastoderm regions and cultured on the VM to assess intrinsic regional adhesive properties. Edge-associated gene expression and cytoskeletal organization were analyzed by *in situ* hybridization and immunohistochemistry.

**Results:**

Explants derived from blastoderm edge regions adhered to the VM at all the newly generated cut edges. However, the adhesive properties became restricted distally during development, with later-stage explants showing loss of attachment at the proximal regions, indicating that adhesive properties are intrinsic to edge regions of blastoderms and become confined distally during development. In explants, the adhesive area extended beyond the narrow distal edge observed in intact embryos. Moreover, the molecular features of edge cells in intact embryos (e.g., edge-associated gene expression and cytoskeletal enrichment) displayed a regionally biased distribution within the expanded edge of the explants.

**Conclusion:**

Blastoderm adhesion to the VM is governed by intrinsic tissue properties that are developmentally regulated and locally specific. Physical attachment to the VM and molecular edge identity are spatially uncoupled, suggesting that epithelial adhesion during early morphogenesis is regulated through coordinated but partially independent molecular and mechanical processes.

## INTRODUCTION

During early avian development, blastoderm expansion over the yolk is essential for proper embryonic morphogenesis [1]. This process, commonly referred to as epiboly, depends on stable attachment of the blastoderm margin to the vitelline membrane (VM), which provides mechanical support and positional anchoring during tissue spreading [2,3]. Disruption of blastoderm-VM adhesion impairs epiboly and is associated with abnormal embryo development, including defects in axis elongation, underscoring the importance of regulated adhesion at the blastoderm edge [4,5].

Embryological studies have shown that attachment to the VM is restricted to the extreme margin of the blastoderm, whereas all other regions of the blastoderm remain unattached [3]. Early experiments demonstrated that this regional specificity reflects the intrinsic adhesive properties of the marginal tissue rather than positional induction: when isolated blastoderm explants were cultured on the VM, adhesion occurred only at the cut edges corresponding to the distal region of the intact blastoderm [5].

Subsequent embryological analyses further supported the view that the blastoderm margin, called edge cells, represents a functionally distinct tissue domain during early development [6,7]. However, it remains unclear how these intrinsic adhesive properties are regulated over developmental time. In particular, whether adhesive properties are strictly confined to the extreme edge or whether their spatial distribution undergoes dynamic remodeling as development proceeds has not been explored.

At the molecular level, several genes enriched at the blastoderm edge, including ribonuclease/angiogenin inhibitor 1 (*RNH1*) and the Wnt-associated regulators dishevelled binding antagonist of beta-catenin 2 (*DACT2*), have been proposed to define edge identity [7,8]. In parallel, cytoskeletal components such as F-actin and vimentin are regionally organized at the blastoderm edge [6,7], suggesting coordinated regulation of tissue mechanics at the embryo margin. These cytoskeletal features are likely to contribute to the structural organization and mechanical properties of the tissue [9,10]. However, it has not been experimentally verified whether these molecular and cytoskeletal features directly correspond to actual sites of physical attachment to the VM.

Here, we investigate the spatial and temporal regulation of blastoderm adhesion to the VM using explant assays and gene expression analysis. We test the hypothesis that the intrinsic adhesive properties of blastoderm tissue become progressively and regionally restricted during development, and that physical attachment to the VM can be spatially uncoupled from molecular features associated with the blastoderm edge.

## MATERIALS AND METHODS

### *Ex-ovo* culture, explant preparation, and fixation

Fertilized Hy-line Brown hens’ eggs were obtained from Bethel farm (Naju, South Korea) and incubated at 38°C to obtain embryos at the desired developmental stages [11]. Eggs were incubated for 12 h or 24 h to obtain embryos at HH stages 3 and 6, respectively. Embryos were cultured *ex ovo* using the modified New culture method [12,13]. To prepare embryos for ex ovo culture, a portion of the eggshell was carefully removed, and thick albumen was discarded while thin albumen was collected and retained. The yolk was transferred into Pannett-Compton solution [14], bisected along the equatorial plane using fine scissors, and the VM was gently separated from the yolk. The isolated VM was stretched over a custom-made glass ring.

**Figure 1:**
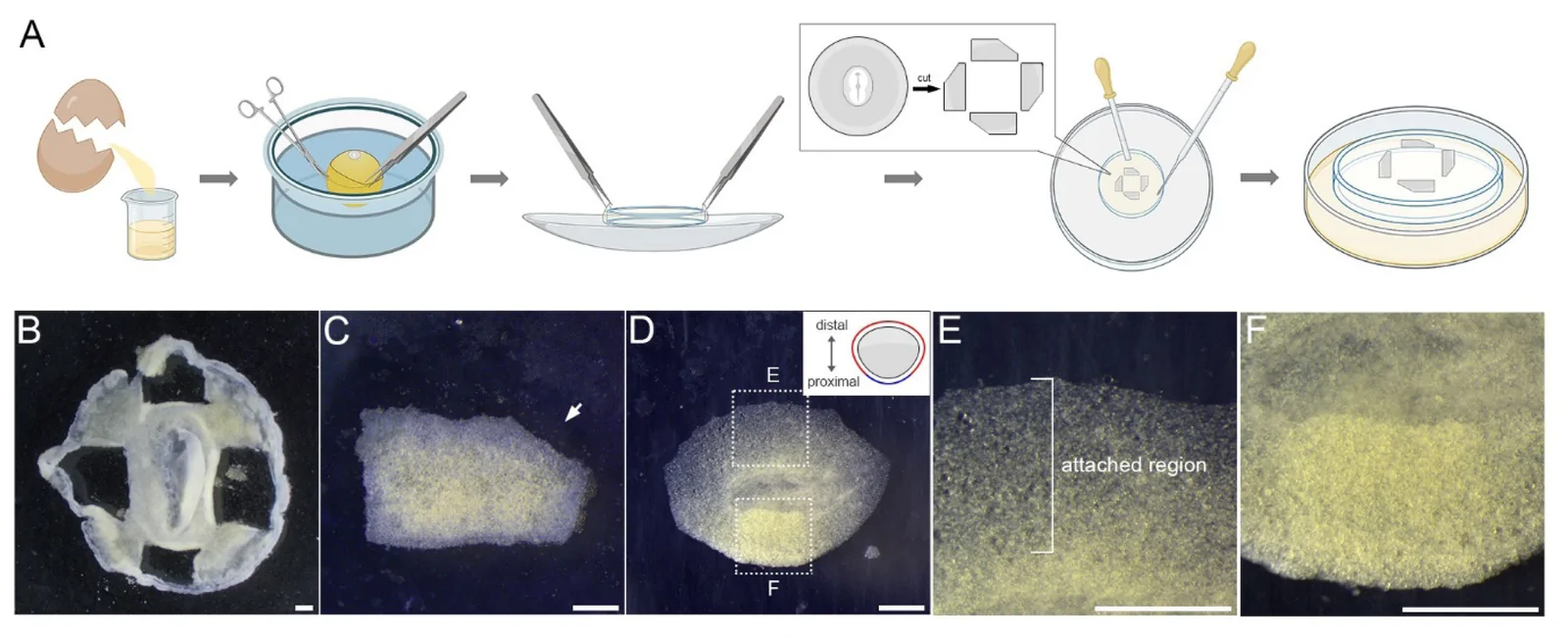
Regional difference in adhesive properties in blastoderm explants. (A) Schematic illustration of the explant assay utilizing the modified new culture method [12]. First, fertilized eggs were opened, and the thin albumen was collected. The yolk was then transferred into a Pannett-Compton solution, and the vitelline membrane (VM) was isolated by bisecting the yolk along the equatorial plane. The isolated VM was then placed onto a watch glass and stretched over a custom-made class ring. Blastoderm explants were excised from non-edge regions of HH stage 6 embryos, generating fragments in which all boundaries are facing free spaces. The explants were then placed onto the prepared VM, after which excess liquid was removed. Finally, the explant-VM-ring complex was cultured in the collected thin albumen in a 35 mm culture dish for 16 h, and attachment to the VM was assessed. (B) A representative image of the HH stage 6 embryo following explant excision. (C) A representative explant immediately after isolation and placement on the VM. A small notch (arrowhead) was introduced to mark the distal side of the blastoderm. (D) An explant after 16 h culture. The inset schematic illustrates the attached (red outline) and unattached (blue outline) regions within the explant. Dashed boxes are shown at higher magnification in panels E and F. (E, F) Higher-magnification views of attached (E) and unattached (F) regions indicated in (D) (n = 17 explants from 14 embryos). The VM-attached region is indicated by a bracket in (E). Scale bars: 500 μm.

For explants, square-shaped tissue fragments were excised from the area opaca except for the edge cells using a bent 26-gauge needle (Figure 1A). After excision, a small notch was introduced at one corner of each explant to identify the original orientation. Explants were then placed onto the VM mounted on the glass ring. Excess liquid was carefully removed, and the glass ring was transferred to a 35-mm Petri dish (35×10 mm; SPL Life Sciences) containing 3 mL of thin albumen to support the VM. Cultures were maintained at 38°C for further incubation. Explants were cultured for 16 h following the experimental conditions established by New, since the primary aim of this study was to investigate the cellular and molecular characteristics associated with the adhesive behavior described in the original assay. Under this same culture duration, we consistently reproduced the characteristic adhesive properties previously reported. Following incubation, adhesion to the VM was assessed by gently lifting the embryos using forceps. Embryos were then fixed overnight at 4°C in 4% paraformaldehyde (PFA) in PBS (pH 7.4) before further analyses.

### Whole-mount *in situ* hybridization

To investigate the spatial expression pattern of genes, whole-mount *in situ* hybridization was conducted as previously described [15]. Fixed embryos were dehydrated in 100% methanol and stored at −20°C overnight. Before whole-mount *in situ* hybridization, dehydrated embryos were rehydrated through a graded methanol series; 75%, 50%, and 25% methanol in PTW (PBS containing 0.1% Tween-20). Embryos were then treated with 20 μg/mL proteinase K diluted in PTW, for various times according to developmental stages; embryos at HH stage 6 were treated for 6 min. Then, embryos were refixed in 4% PFA containing 0.1% glutaraldehyde at room temperature for 20–30 min. Samples were subsequently equilibrated in hybridization solution and then incubated with digoxigenin-labeled RNA probes specific for the genes of interest. The probes used were *RNH1* (ChEST73n20) and *DACT2* [16]. Hybridization was carried out at 70°C for at least 16 h. Following hybridization, embryos were washed multiple times with TBST (Tris-buffered saline containing 0.1% Tween-20) and incubated in blocking buffer consisting of 5% heat-inactivated goat serum and 1 mg/mL bovine serum albumen (BSA) for 3 h at room temperature. Embryos were then incubated with anti-digoxigenin alkaline phosphatase-conjugated antibody (1:5,000; Roche; Cat. No. 11093274910) diluted in blocking buffer at 4°C for at least 16 h. After extensive washing with TBST, color development was performed using NBT/BCIP substrate solution until a signal was detected.

### Immunohistochemistry

For immunohistochemistry, fixed embryos were washed in PBST (PBS containing 0.1% Triton X-100) to remove residual fixative. To facilitate antibody penetration, embryos were treated with 1% Triton X-100 in PBS for 20 min. Samples were then blocked in blocking solution consisting of 5% normal goat serum and 0.02% thimerosal in PBST for 3 h at room temperature. Rabbit anti-vimentin (Abcam; Cat. No. ab92547) was diluted (1:200) in blocking solution and applied to embryos for 2 days at 4°C. After multiple washes with 0.1% PBST, embryos were incubated with goat anti-rabbit IgG (H+L)-Alexa Fluor 594 (Abcam; Cat. No. ab150080) diluted 1:200 in blocking solution at 4°C for at least 16 h. Following extensive washing with 0.1% PBST, nuclei and F-actin were counterstained with Hoechst 33342 (1;1,000; Abcam; Cat. No. ab228551) and phalloidin (1:1,000; Abcam; Cat. No, ab176753) diluted in PBS for 40 min. Samples were then washed in PBS, mounted on glass slides using an anti-fade fluorescence mounting medium (Abcam; Cat. No. ab104135), and covered with a coverslip before imaging.

### Microscopy, image acquisition, and analysis

Embryo morphology before and after culture, as well as gene expression patterns following whole-mount *in situ* hybridization, were examined using a stereomicroscope (Leica S9D; Leica Microsystems). Immunohistochemical staining was imaged using a confocal laser scanning microscope (Leica STELLARIS 5; Leica Microsystems). Obtained images were further analyzed using Fiji (ImageJ) software [17].

### Statistical analysis

Statistical analyses were performed using GraphPad Prism (ver. 6.0; GraphPad Software). Differences in explant area were analyzed using an unpaired two-tailed Student’s t-test with Welch’s correction. A p-value<0.05 was considered statistically significant.

## RESULTS

### Confirmation of intrinsic adhesive properties of blastoderm edge tissue

Throughout this study, the terms “distal” and “proximal” are used relative to the center of the blastoderm, with “distal” referring to regions closer to the blastoderm margin and “proximal” referring to regions towards the interior of the blastoderm. Previous studies reported that the adhesive property of chick blastoderm tissue to the VM is restricted to the distal regions of the blastoderm [5]. However, the cellular and molecular characteristics underlying this regional restriction remain poorly understood. We therefore re-examined this classical observation by isolating blastoderm explants from HH stage 6 embryos that did not include the original blastoderm edge (Figures 1A–1C).

Following 16 h culture, attachment was observed at explant edges corresponding to regions originally adjacent to the blastoderm edge (distal region), whereas edges derived from the proximal region consistently remained unattached or barely attached (Figure 1D). If adhesive ability can be acquired when cells are facing free space, all edges of the explants would be expected to attach to the VM. However, this was not observed, indicating that adhesive properties are intrinsically specified within defined blastoderm regions. This result is consistent with previous observations reporting intrinsic adhesive properties of blastoderm tissue [5].

Morphologically, the attached regions appeared more translucent than the unattached regions, suggesting differences in tissue thickness or cellular organization (Figures 1E, 1F; n = 17 explants from 14 embryos). Notably, this morphology of attached regions resembled previously described edge cell architecture [7], suggesting that acquisition of adhesive properties may be accompanied by region-specific morphological remodeling. It is noted that, unlike intact blastoderm, where only the distal-most cells (2–4 cells) attach to the VM, the attachment region was much expanded in explants (bracket in Figure 1E).

### Spatiotemporal restriction of adhesive properties in the blastoderm edge

**Figure 2:**
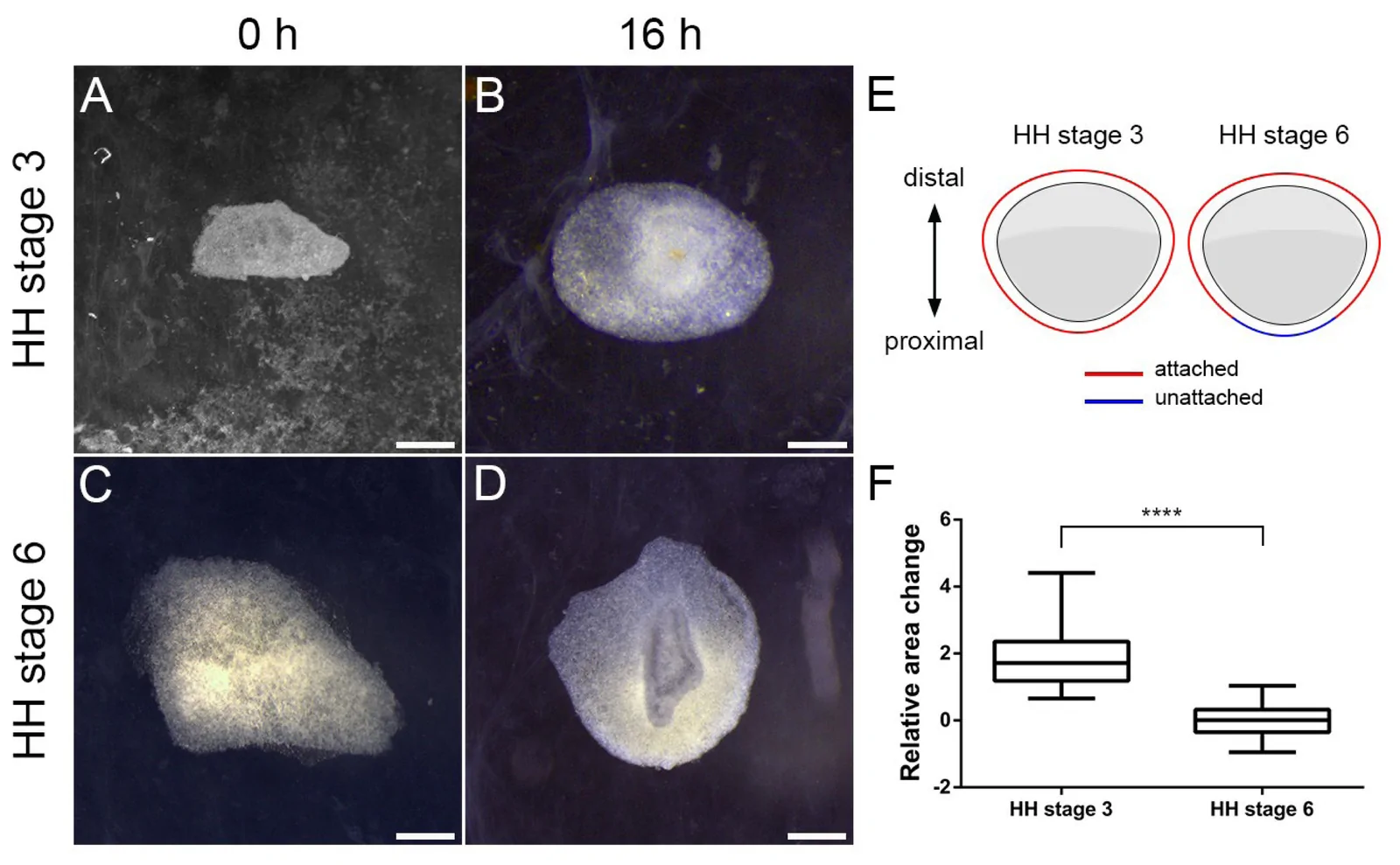
Developmental restriction of adhesive property in the blastoderm explants. (A–D) Explants from HH stage 3 (A, B) and HH stage 6 (C, D) embryos immediately after isolation (0 h; A, C) and after 16 h culture (B, D). (E) A schematic illustration summarizing the attachment outcomes after 16 h culture, showing attached (red outline) and unattached (blue outline) regions. (F) Relative area changes after 16 h culture, calculated as (area at 16 h – area at 0 h) / (area at 0 h). Data are shown as box and whisker plots showing the median (center line), interquartile range (box), and minimum to maximum values (whiskers) (HH stage 3, n = 20 explants from 10 embryos; HH stage 6, n = 45 explants from 25 embryos). Scale bars: 500 μm. Statistical significance was assessed using an unpaired two-tailed Student’s t-test with Welch’s correction. **** p<0.0001.

To examine how adhesive properties change during development, we performed the same explant assay using early embryos (HH stage 3) and compared the results with those obtained from HH stage 6 embryos (Figures 2A–2E). As a result, a regional difference in attachment was observed. Explants derived from HH stage 3 embryos consistently exhibited attachment along all cut edges (Figures 2B, 2E). In contrast, explants from HH stage 6 embryos showed attachment restricted along edges corresponding to the original distal blastoderm region, whereas edges derived from proximal regions consistently remained unattached (Figures 2D, 2E). In addition, the area increase in explant during culture were significantly greater in HH stage 3 explants than in HH stage 6 explants (Figure 2F; HH stage 3, n = 20 explants from 10 embryos; HH stage 6, n = 45 explants from 25 embryos; unpaired two-tailed Student’s t-test with Welch’s correction, **** p<0.0001), indicating that tissue spreading capacity, together with adhesive behavior, becomes developmentally restricted. Together, these results demonstrate that cells’ adhesive ability, which appears when facing free space, is broadly distributed at earlier stages but becomes spatially restricted at later stages.

### Expression of edge-associated genes in regions acquiring adhesive properties

To determine whether the regions of the explant that acquire adhesive properties also exhibit molecular features of the blastoderm edge, we investigated the expression patterns of two genes known to be enriched at the blastoderm edge, *RNH1* and *DACT2* [7,8], by whole-mount *in situ* hybridization. Explants derived from HH stage 6 embryos were cultured for 16 h and subsequently analyzed.

**Figure 3:**
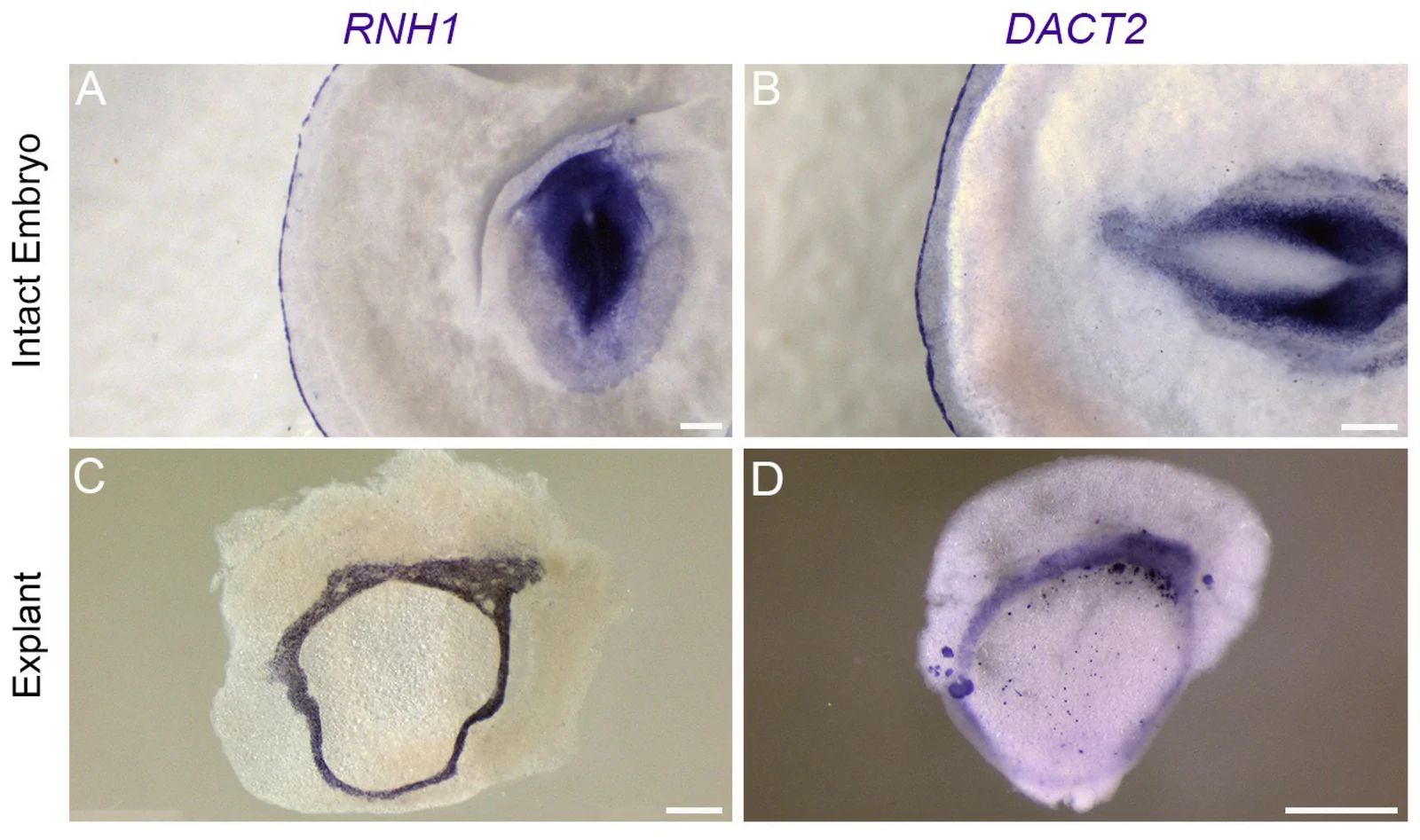
Expression patterns of edge-associated genes in intact embryos and explants. (A, B) Whole-mount *in situ* hybridization showing expression of *RNH1* (A) and *DACT2* (B) in intact HH stage 6 embryos. (C, D) Expression of *RNH1* (C) and *DACT2* (D) in explants. derived from HH stage 6 embryos after 16 h culture (*RNH1*, n = 7 explants from 3 embryos; *DACT2*, n = 6 explants from 4 embryos). For explants, the top-to-bottom direction corresponds to the distal-to-proximal axis. Scale bars: 500 μm.

As a result, the expression levels were not uniform across explants; a distal-to-proximal gradient was consistently observed, with stronger and wider signals concentrated toward the attached distal regions, while lesser and narrower expression was observed in the unattached (or barely attached) regions. Both *RNH1* and *DACT2* were localized predominantly in the inner regions of the explants (Figures 3C, 3D; *RNH1*, n = 7 explants from 3 embryos; *DACT2*, n = 6 explants from 4 embryos), rather than in the outermost cells as observed in intact blastoderm (Figures 3A, 3B). Notably, these gene-expressing regions coincided with the inner boundary of the expanded attached area of the explants, consistent with previous findings that their expression is enriched in the proximal cells of the edge cells of the intact embryos [7]. These results suggest that *RNH1* and *DACT2* are associated with the acquisition of adhesive properties but do not exclusively mark their outermost position in the tissue.

### Regional distribution of cytoskeletal markers within expanded adhesive zones

**Figure 4:**
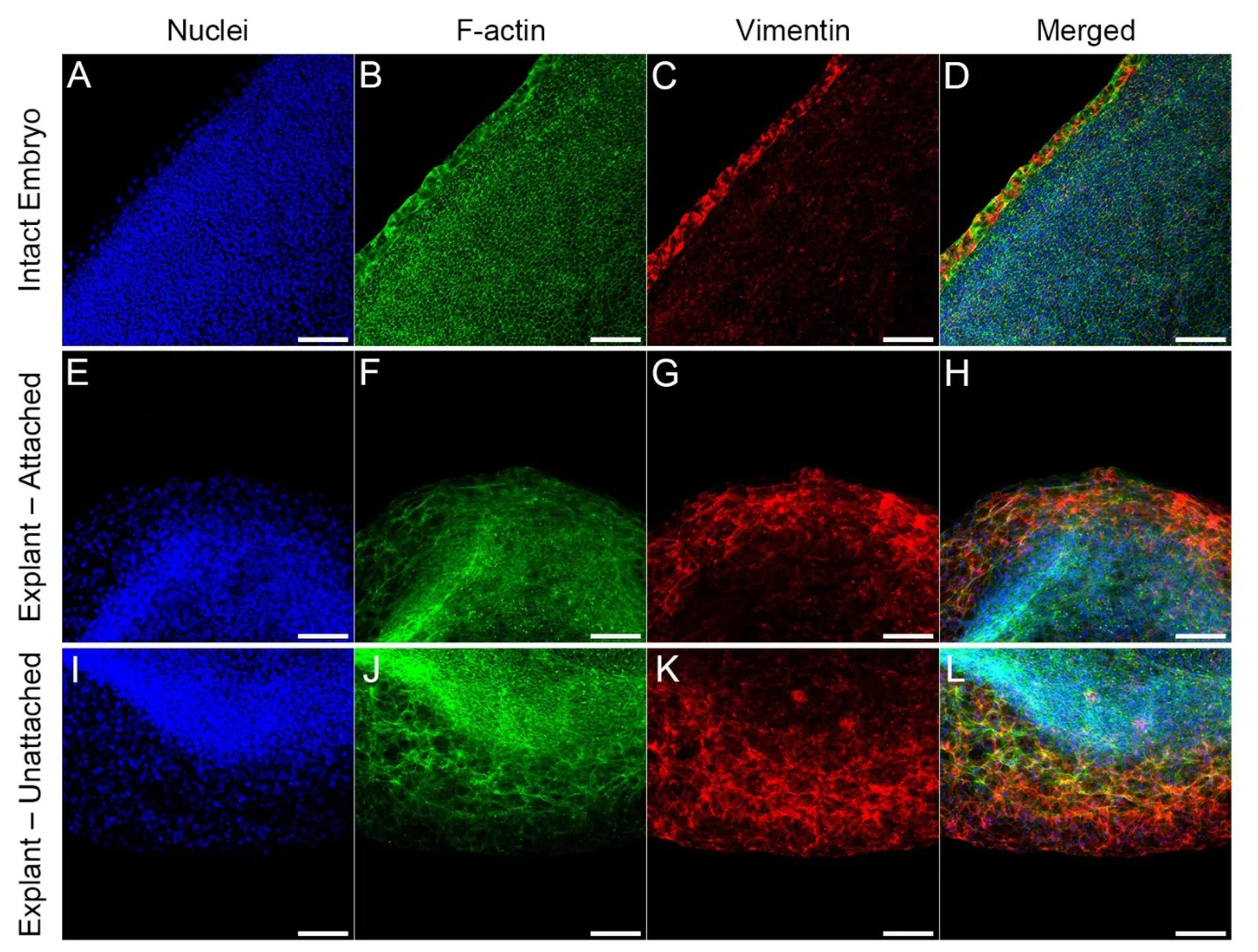
Immunohistochemical images showing spatial organization of cytoskeletal markers within expanded adhesive domains. (A–D, E–H, I–L) Nuclei, F-actin, vimentin, and merged images in (A–D) intact embryos (n = 2 embryos), (E–H) VM-attached regions of explants, and (I–L) VM-unattached regions of explants (n = 2 explants from 2 embryos). Scale bars: 100 μm. VM, vitelline membrane.

To further characterize the molecular features of adhesive zones, we compared the spatial distribution of F-actin and vimentin between intact HH stage 6 embryos and explants cultured for 16 h (Figure 4). In intact embryos, strong F-actin and vimentin signals were confined to a narrow distal edge region consisting of 2–4 cell layers (Figures 4B–4D). This region exhibited broadened nuclear morphology, consistent with previously described edge cell architecture (Figures 4A; n = 2 embryos) [6,7,18]. In explants, both the domain exhibiting broadened nuclear morphology and the region displaying strong cytoskeletal signals were markedly expanded compared to intact embryos (Figures 4E–4L). This expansion was evident in both attached and unattached regions of explants. Within these expanded regions, however, the spatial distribution of markers was not uniform. F-actin was predominantly enriched in the inner regions of the tissue (Figures 4F, 4J), whereas vimentin was preferentially localized in the outer regions (Figures 4G, 4K; n = 2 explants from 2 embryos). These results suggest that, although the explants exhibit similar edge-associated molecular features, cytoskeletal and adhesion-related components remain spatially segregated within the expanded domain.

### Summary of adhesive and molecular features in intact embryos and explants

**Figure 5:**
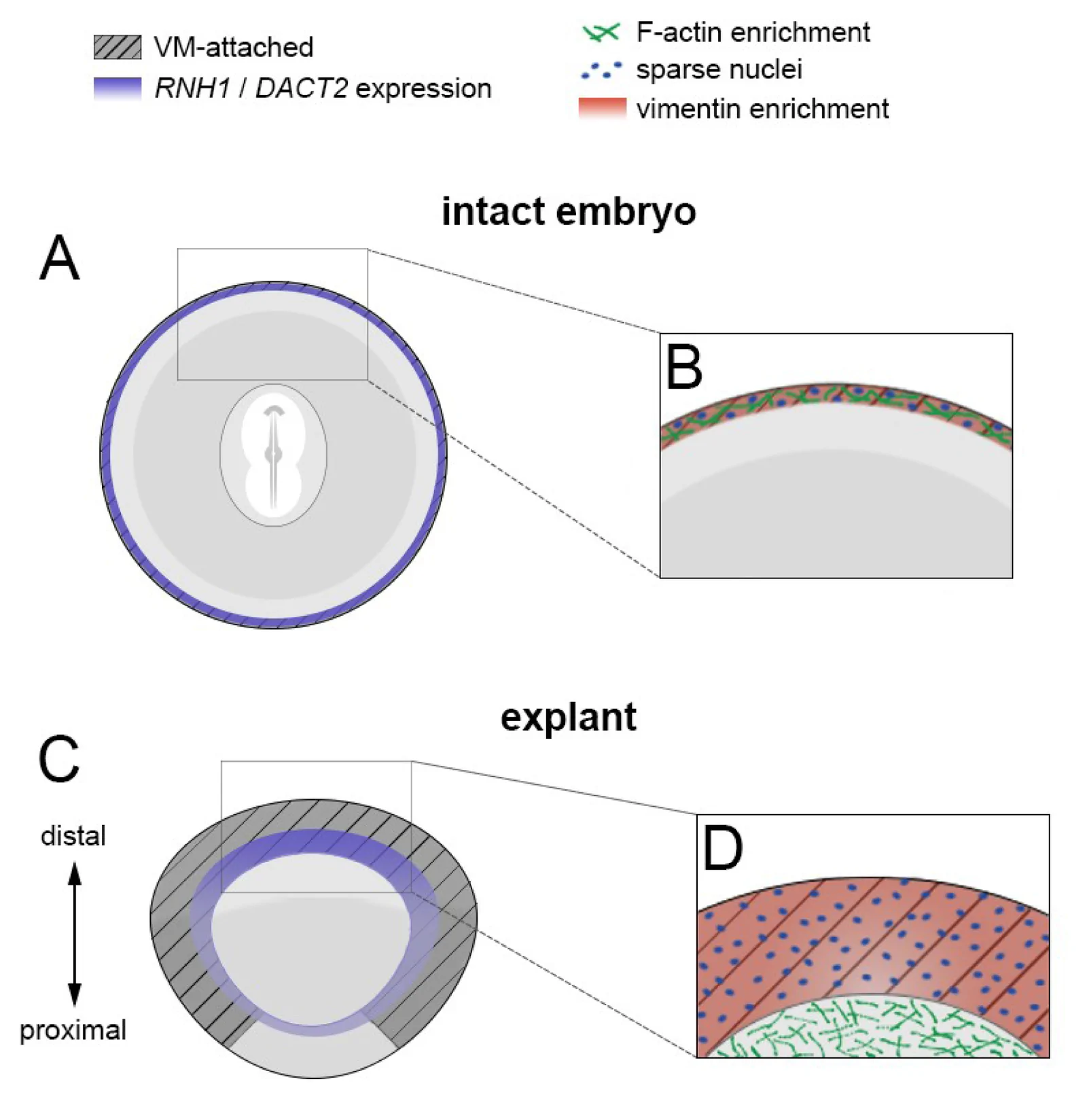
A schematic summary of adhesive and molecular features in intact embryos and explants at HH stage 6. (A) An intact embryo showing the VM-attached distal region and the expression patterns of *RNH1* and *DACT2*. (B) A higher-magnification view of the boxed region in (A), showing F-actin enrichment, sparse nuclei, and vimentin enrichment. (C) An explant showing the expanded VM-attached region and the corresponding expression of *RNH1* and *DACT2*. (D) A higher-magnification view of the boxed region in (C), showing F-actin enrichment, sparser nuclei, and vimentin enrichment. For clarity, only regions relevant to the attached region are illustrated. VM, vitelline membrane.

To integrate these findings, we generated a schematic summary illustrating the spatial relationship between adhesive properties and molecular features in intact embryos and explants at HH stage 6 (Figure 5). In intact embryos, VM attachment is restricted to the distal-most region, where edge-associated genes (*RNH1* and *DACT2*) are expressed together with enriched F-actin. Nuclei are sparsely distributed within this region (Figures 5A, 5B). In explants, the VM-attached region is markedly expanded; however, the expression of *RNH1* and *DACT2* is not uniformly distributed and is instead localized toward the inner boundary of the expanded adhesive domain (Figure 5C). Within this expanded region, cytoskeletal and adhesion-related components remain spatially segregated, with F-actin enriched in inner regions and vimentin preferentially localized toward the outer regions. Nuclei are sparsely distributed across the expanded adhesive domain (Figure 5D). Together, these findings indicate that although adhesive properties expand in explants, molecular features are reorganized rather than uniformly redistributed.

## DISCUSSION

In this study, we investigated the spatial and developmental regulation of the adhesive property of the blastoderm to the VM in chick embryos. Using explant assays, we confirm the observation in the previous study by New [5] that adhesive properties are intrinsically confined to the distal region of the blastoderm, rather than being acquired by a cell’s position. In addition, we demonstrate that these adhesive properties become restricted during development. Explants derived from early-stage embryos exhibited attachment along all cut edges, whereas later-stage embryos showed adhesion confined to distal regions. Because all explant boundaries were newly generated, this patterned restriction cannot be explained by positional effects. Instead, it reflects developmental regionalization of intrinsic tissue properties. Such maturation-dependent restriction may arise from progressive change of epithelial organization, mechanical tension, or extracellular matrix interactions during blastoderm development [19].

Interestingly, the adhesive zone was broader in explants than in intact embryos, by extending into more proximal regions [6,7]. However, this expanded adhesive zone did not uniformly correspond to the molecular features of the blastoderm edge. Although edge-associated genes and cytoskeletal and adhesion-related markers were broadly detected within explants, their spatial organization remained biased, with distinct enrichment patterns. This spatial compartmentalization indicates that physical attachment to the VM and internal cytoskeletal remodeling are coordinated yet partially independent processes [20]. Although the precise role of these accumulations remains unclear, they may contribute to processes such as collective cell migration, mechanical stabilization, or structural remodeling similar to their proposed functions at the edge of intact embryos [2,3,7]. While validating these functional roles requires further investigation in future studies, our findings provide a valuable descriptive foundation for the spatial segregation of these cytoskeletal components.

## CONCLUSION

In summary, our data show that blastoderm adhesion to the VM is governed by intrinsic, developmentally regulated, and regionally patterned tissue properties rather than simple positional cues at free edges. Importantly, physical attachment to the VM and the molecular features of the blastoderm edge are not strictly co-localized. This spatial uncoupling between adhesion and edge identity suggests that epithelial adhesion during early morphogenesis is controlled by multiple, partially independent regulatory modules integrating signaling pathways, cytoskeletal organization, and tissue mechanics.
